# Insights Into the Research Landscape of Biomedical Physics: A Bibliometric Inquiry

**DOI:** 10.7759/cureus.63764

**Published:** 2024-07-03

**Authors:** Vinoj M N, Bindu R G, Dhanyasree A R, Beenamol T, Jobin Jose

**Affiliations:** 1 Department of Physics, St. Peter's College, Kolenchery, Kolenchery, IND; 2 Department of Physics, NSS College, Nilamel, Kollam, IND; 3 Department of Library Science, Iqbal College of Arts and Science, Thiruvananthapuram, IND; 4 Department of Library Science, Mar Ivanios College, Thiruvananthapuram, IND; 5 Department of Library Science, Marian College Kuttikkanam Autonomous, Kuttikkanam, IND

**Keywords:** bibliometric review, vosviewer, biblioshiny, bibliometric analyses, biomedical physics

## Abstract

Biomedical physics is the interdisciplinary field that links the scientific concepts in physics to the practice of medicine and biology, in an effort to understand biological processes, help in the development of medical technologies, to improve human health. This bibliometric study investigates the interdisciplinary field of biomedical physics, which integrates the principles of physics with biological and medical sciences to develop innovative diagnostic and therapeutic technologies. Utilizing the Web of Science database for bibliographic data collection, the analysis employs advanced bibliometric software tools, including Biblioshiny and VOSviewer, to comprehensively map the research landscape. Our findings delineate the annual scientific production, highlighting growth trends and identifying the most influential authors and key publication venues in the field. A thematic analysis reveals prevailing research topics and the evolution of scientific interests over time, providing insights into the shifting focus areas within biomedical physics. The factorial analysis goes further to clarify the conceptual structure of the discipline by providing a topological image of how the different research areas are involved. It helps to recognize topical fields and the possibility of the topicalization of other subjects. Keyword co-occurrence assumes the leading themes and measures the value of the topology. Meanwhile, bibliographical information defines the authors’ network, and co-citation analysis identifies the critical authors’ pool. The last points to the topic dependence and the network of research collaboration on a global scale. As a result, a survey identifies the deficits and rules of recommendations for the further development of research. It adds practical implications that are necessary for the development and identifies influences for popularization that it might have in the future.

## Introduction and background

Biomedical physics is a striking combination of medicine, biology, and physics that extends the envelope of human health cognizance and healing by integrating novel physical science-derived methods [1]. This interdisciplinary scientific field uses physics to intricate models rooted within biology and medicine and has had a significant impact on diagnoses and treatment technology, advancing such outdated regimes and diagnostic schemes [2,3]. Biomedical physicists help with medical research, design medical equipment, and work on ways to detect and treat diseases and alleviate other causes, hence lecturing/joining forces with experts in distinct scientific disciplines to partake in forming character-controlled patient control strategies [4,5]. Medical physics is an ocean of interdisciplinary involvement, diffusing physicians and biologists, engineers, and computer scientists - a modulation that is critical for having an impact on societal change [6]. Integrating physicists into distinct technological entrepreneurship holds a secret, according to experts - the unremitting flow of expertise amid actors with unhappy options has hastened the rate of limits of human growth [5]. Physicists' science and technology transformation feed off of one another; the progress of each advances diagnostic and clinical utilities, as far as textbooks prove profitable out of laboratory types and substitute patient influences, gradually improving energy and increasing discriminative energy and knowledge of life phenomena from the molecule level to systems [7].

Biomedical physics is a vast, growing field with increasing roots and progression. The continuous broadening and reach of the scope demand a systematic inquiry into the research ecosystem of biomedical physics [1,8]. With bibliometrics, it is possible to establish a strong analytic framework that allows for cutting through the publication behavior of researchers in terms of areas, trends, citation functions, research networks, academic partnerships, and more [9-13]. Understanding the history of the current field, emerging themes, and the connections across areas of the world and countries will highlight more traceable paths for the future’s innovation and disciplinary confront of this essential area of care [14,15].

To provide more context, bibliometrics is a statistical method used to analyze books, articles, and other publications, offering insights into research trends, authorship patterns, and citation networks. Tools like Biblioshiny (University of Naples Federico II, Italy) and VOSviewer (Leiden University, The Netherlands) are instrumental in conducting these analyses. Biblioshiny is a web-based application that allows users to perform comprehensive bibliometric analysis, generating detailed visualizations of research dynamics over time. VOSviewer, on the other hand, specializes in creating network visualizations, helping to map the relationships between authors, institutions, and research themes. The Web of Science collection is a robust database that provides access to a vast array of scholarly articles, serving as a foundational resource for bibliometric studies.

The present bibliometric study is dedicated to the interdisciplinary domain of biomedical physics. Its analysis is based on the multiple functionalities of Biblioshiny and VOSviewer, which are used to investigate the bibliographic data from the Web of Science collection. With the help of these innovative tools, we investigate the specific patterns of research production, collaboration, and thematic evolution within the field of biomedical physics. Given that Biblioshiny can be used to retrieve and visualize the dynamics over time [16,17] and VOSviewer is used to create comprehensive network visualizations of the authors, institutions, and research themes, this combination is optimal for charting the growth and critical nodes of various trends [18-21]. In this way, we aim to present the most critical contributors to the field and the prominent journals that produce the major discourse. The supplemental benefit of this methodological approach lies in the identification of the most critical areas of research and emergent trends [22]. It explores how technological advancements co-engineered theoretical development within the pretty novel but rapidly expanding domain of biomedical physics. Overall, the present bibliometric analysis unpacks the most successful research trends and their dynamics, applicable for considering the future development of the field. In this regard, it provides a starting ground for other researchers, policymakers, and educators to make informed decisions regarding the most crucial sectors, areas of collaboration, and educational areas for further development. Ultimately, this study offers a strategic guide for developing and ensuring the growth of biomedical physics as a major domain for the improvement of healthcare and technology.

Research questions

The research questions (RQs) are as follows: RQ1: What are the core research themes in biomedical physics, and how have they evolved? RQ2: Who are the key contributors in the field of biomedical physics? RQ3: What are the publication trends in biomedical physics research, including publication counts, and journal distribution? RQ4: How does international collaboration influence the research output and citation impact of publications in biomedical physics? RQ5: What are the unrepresented areas, research gaps, and potential interdisciplinary opportunities in the field of biomedical physics?

## Review

Materials and methods

Web of Science core collection was chosen as this study's primary bibliographical data source because it covers a broader range of quality journals compared to other databases [23]. The publications were retrieved using the keyword "biomedical physics." There were no language restrictions; only journal articles, conference papers, and book chapters were considered. A total of 1,222 documents were collected from 503 different sources between 1990 and 2024. We excluded reviews, editorials, books, short notes, and surveys in the second phase. Documents included are articles, conference papers, and book chapters. The findings were stored as a "CSV" file, and bibliometric analysis was performed on the data using the Biblioshiny software and VOSviewer. The main aspects of this investigation are summarized in Table 1.

**Table 1:** Main information of the investigation

Description	Results
Main information about data	
Timespan	1990-2024
Sources (journals, books, etc.)	503
Documents	1222
Annual growth rate %	9.37
Document average age	10.5
Average citations per doc	48.41
References	49951
Document contents	
Keywords plus (ID)	3448
Author's keywords (DE)	3041
Authors	
Authors	5439
Authors of single-authored docs	116
Author collaboration	
Single-authored docs	125
Co-authors per doc	5
International co-authorships %	26.68
Document types	
Article	1087
Article; book chapter	1
Article; early access	6
Article; proceedings paper	128

The study is based on a comprehensive dataset consisting of materials from 1990 to 2024 and is used to conduct a bibliometric analysis of the field of biomedical physics. The dataset includes a total of 1,222 documents drawn from 503 variously ranked journals, books, and other sources relevant to the given field, thus reflecting in-depth research activities among scientists. Moreover, the data reflect a substantial amount of work on biomedical physics, with the average age of documents being 10.5 years. This shows the academic breadth of research, covering both historical and modern approaches to the problem. In addition, the dataset has an average annual growth rate of 9.37%, indicating a growing interest and the continuous development of biomedical physics research over the years. Each document, on average, has a total of 48.41 citations, thus pointing to the considerable impact of research included in the dataset. Moreover, the dataset contains a total of 49,951 references, indicating a highly developed reference base for research.

Results and discussion

Annual Scientific Production

**Figure 1:**
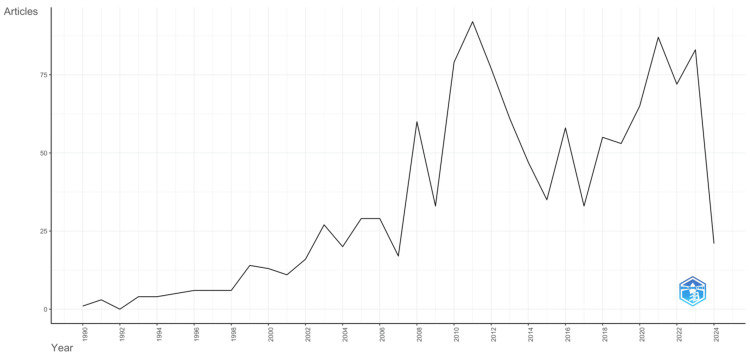
Annual scientific production

The number of published articles in biomedical physics has significantly increased from 1990 to 2024, as presented in Figure 1. In the first few years, relatively few publications are produced, with the frequency then increasing steadily. This trend reveals a growing reservoir of interest and sustained development in the broad field of study and interdisciplinary specialty of the topic. Furthermore, this graph shows a recurring pattern of variability in the annual production, which might indicate the natural rhythm of alternation in scientific inquiry in which various levels of hybrid funding, the phase of technological development, or human health and welfare policies may be involved. Owing to the graph, it is possible to notice two peaks in publication numbers occurred around 2010 and 2020, so there have been some seminal moments that have caused an outburst in research and publications in the biomedical physics field. There could be a number of explanations for this; the most important is most likely significant progress in medical technology, changes in pandemic priorities, or enhanced healthcare. Nevertheless, after 2020 and most recently, there has been a significant decrease in the volume of productivity. This finding could signify a reorientation of research interest or the repercussions of complex external factors like economic issues or the COVID-19 pandemic. This graph is one element of bibliometric analysis because it reveals not just the momentum in output over time but also the dynamic nature of change in this wide field.

Most Relevant Sources

**Figure 2:**
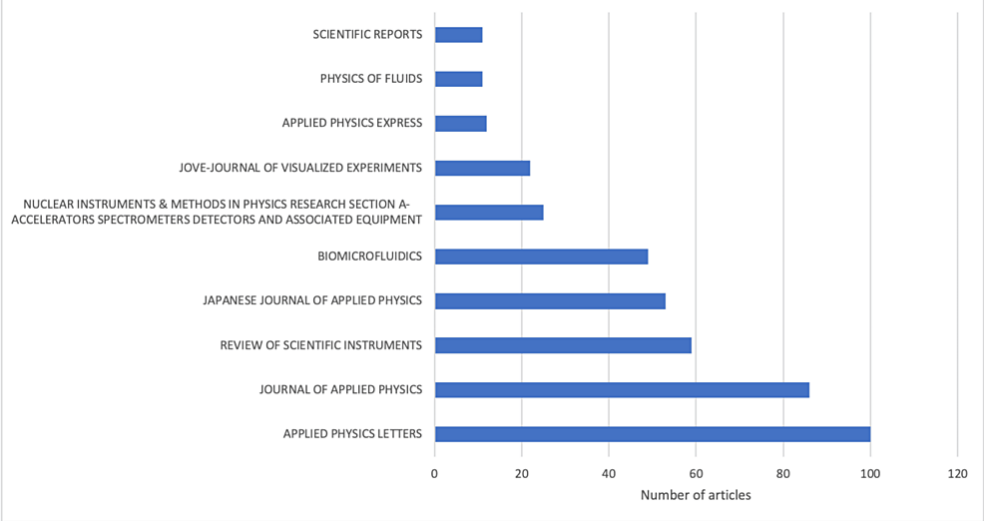
Most relevant sources

In the realm of biomedical physics, the dissemination of research findings through scholarly publications is pivotal to the advancement of knowledge and innovation. The bibliometric analysis presented here focuses on identifying the most relevant sources that have been instrumental in shaping the discourse and progress within the field. These sources, recognized by their frequency of article publications, serve as key outlets for the latest research developments, experimental findings, and theoretical advancements in biomedical physics. Figure 2 presents "Applied Physics Letters" as the leading source with 100 articles, signifying its influential role in the scientific community. It is followed by the "Journal of Applied Physics," contributing 86 articles, and the "Review of Scientific Instruments," with 59 articles, each playing a significant part in the applied research landscape. Publications like the "Japanese Journal of Applied Physics" and "Biomicrofluidics," with 53 and 49 articles, respectively, emphasize the global contribution and the specialized domains that biomedical physics encompasses. Other notable sources include "Nuclear Instruments & Methods in Physics Research Section A," "JOVE-Journal of Visualized Experiments," "Applied Physics Express," "Physics of Fluids," and "Scientific Reports," each with a meaningful presence evidenced by their article count. The diversity of these journals reflects the broad scope of biomedical physics and underlines the importance of interdisciplinary approaches within the field.

Most Relevant Authors

**Figure 3:**
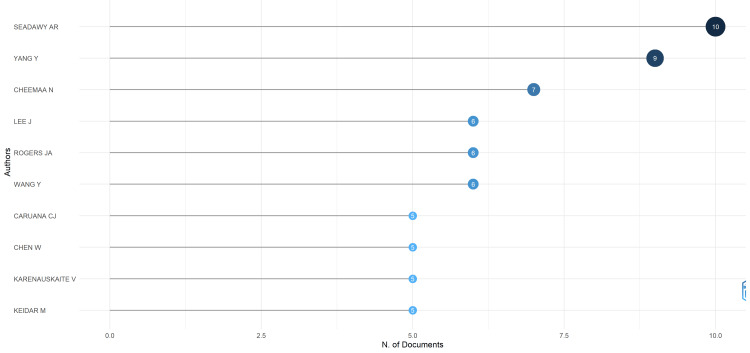
Most relevant authors

Figure 3 shows the most relevant authors in the field of biomedical physics and presents an intriguing snapshot of those whose contributions have been prolific in terms of published articles. At the forefront is Seadawy AR, with a remarkable record of 10 articles to their credit, reflecting a substantial individual or leading group contribution to the body of research. The fractionalized article count, which accounts for co-authorship by dividing the credit for each article among its authors, still places Seadawy AR at the top with a fractionalized count of 3.45, indicating a significant role in the collaborative research efforts. Following are authors Yang Y and Cheemaa N, with nine and seven articles, respectively, and with fractionalized counts of 1.24 and 2.28, showing a strong presence in the field, both in terms of solo and collaborative works. The list also includes Lee J, Rogers JA, and Wang Y, each contributing six articles. Although the article counts are similar, the fractionalized scores differ, implying different degrees of collaboration: Rogers JA appears to manifest a higher rate of solo or primary authorship, while the remaining authors appear more collaborative. Caruana CJ, Chen W, Karenauskaite V, and Keidar M, each with five articles, further intensify the context between the number of articles written by authors in biomedical physics and the diversity and intensity of their collaboration. The fractionalized nature of article counts presented in the table above provides additional clarifications about the collaboration and the contribution of these authors to articles authored by multiple individuals. All of these writers are eminent in the bibliometric evaluation of the number of articles produced and in terms of the collaboration that appears to be a dominant paradigm enhancing the research landscape in biomedical physics.

Trend Topics

**Figure 4:**
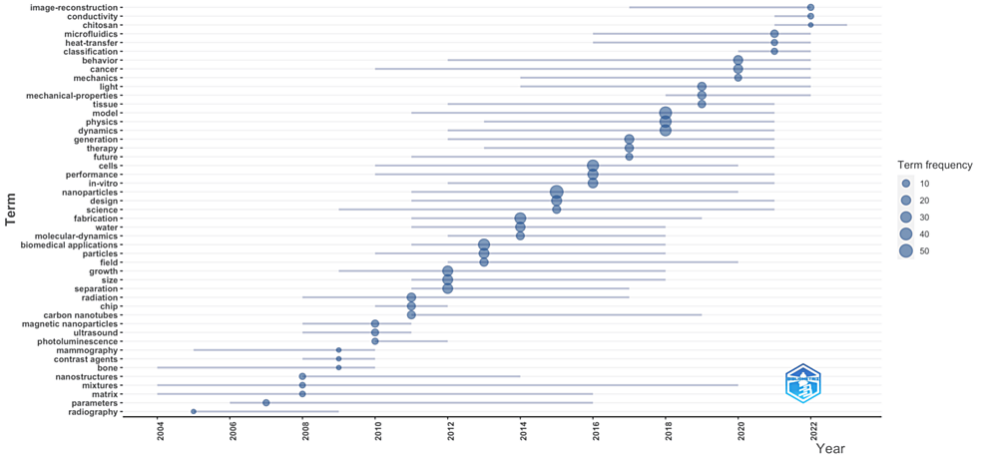
Visualization of trend topics

The trend topics in Figure 4 illustrate the shifting focus of the field over a timeline from 2004 to 2022. During the early years, topics such as “microfluidics,” “mechanics,” and “image reconstruction” dominated the heatmap, indicating a substantial focus on creating ways of watching and controlling biological processes. In addition, the high use of terms like “heat-transfer” and “conductivity” during these years displays the need to understand vital physical phenomena within biological systems, paving the way for new diagnostic and therapeutic technologies. The moderate point of the timeline is marked by a move toward materials science and nano-scale research, with terms like “nanoparticles,” “carbon nanotubes,” and “magnetic nanoparticles” peaking in frequency. This is consistent with the incorporation of nanotechnology into biomedical physics, making new drug delivery systems, better imaging techniques, and groundbreaking new treatments feasible. The final quarter of the timeline contrasts sharply with other periods. This is evidenced by biomedical applications’ relative importance, which suggests that research is becoming more maturate, either by becoming more focused on clinical and healthcare problems or by becoming more directly linked to previously theoretical matter. “ultrasound” and “contrast agents” and “radiography” eventually win out, indicating that effort is being made to enhance current imaging methods and devise better diagnostic instruments. These theme's emergence and rise to prominence are a reflection of the considerable progress that has been made in medical imaging over this period, as well as the additional areas in which further studies may contribute to the improvement of non-invasive diagnostics. Hence, these trends not only reflect the progression of research themes in biomedical physics but also the field’s devotion to technological change and healthcare concerns.

Thematic Map

**Figure 5:**
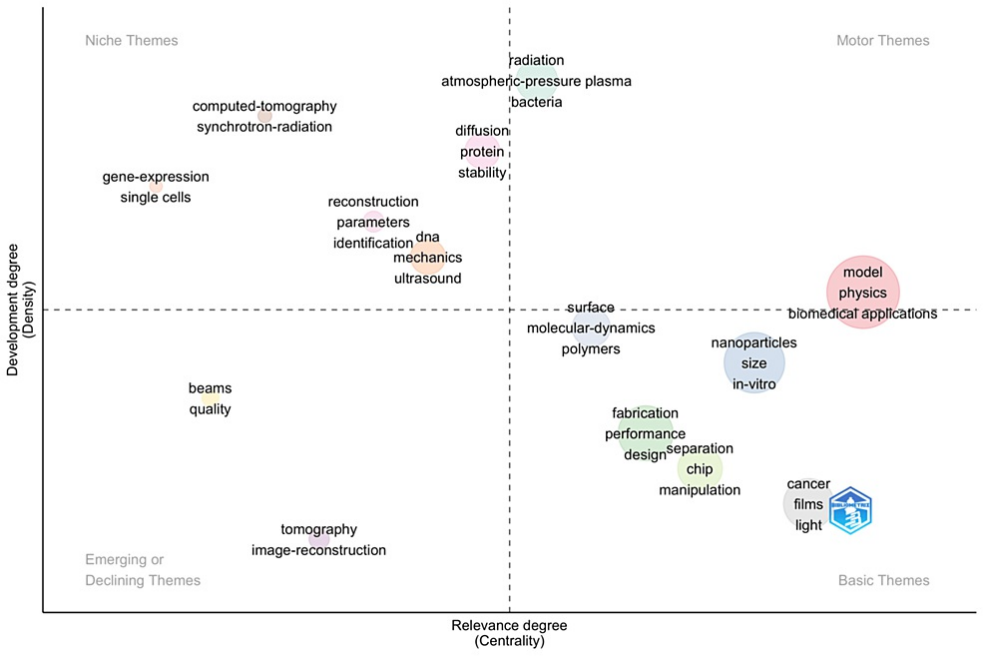
Thematic visualization of keywords

The thematic map displayed in Figure 5 provides a structured view of the field's thematic orientation and the relative centrality and development of different research topics. The visualization places themes in four quadrants, representing basic themes, niche themes, emerging or declining themes, and motor themes. One can recognize the basic themes, niche themes, and motor themes. Basic themes, such as cancer, film, and light, are developed in the sense that they have an ample base of research, yet they are also developed in the sense that they were found to be in the center of the field of biomedical physics. Niche themes, such as computed tomography and synchrotron radiation, are developed to a great extent, but the centrality is low in the sense that these are specialized themes; although advanced, they attract a relatively smaller portion of the research community. Other emerging or declining themes found on the map are tomography and image reconstruction, which are seen as less developed and less central themes that denote areas that are either increasing to be a new trend area in research frontiers or decreasing as the area is about to develop. The most dynamic quadrant contains motor themes, which are both highly developed and central to biomedical physics. Keywords like "model," "physics," "nanoparticles," and "biomedical applications" suggest that these are driving forces in the field, propelling forward research and collaboration. These motor themes are likely where significant innovations and cross-disciplinary interactions are concentrated, shaping the current and future landscape of biomedical physics research. The thematic map thus not only reflects the current state of biomedical physics research but also provides insights into the strategic domains likely to influence its future trajectory.

Factorial Analysis: Conceptual Structure Map

**Figure 6:**
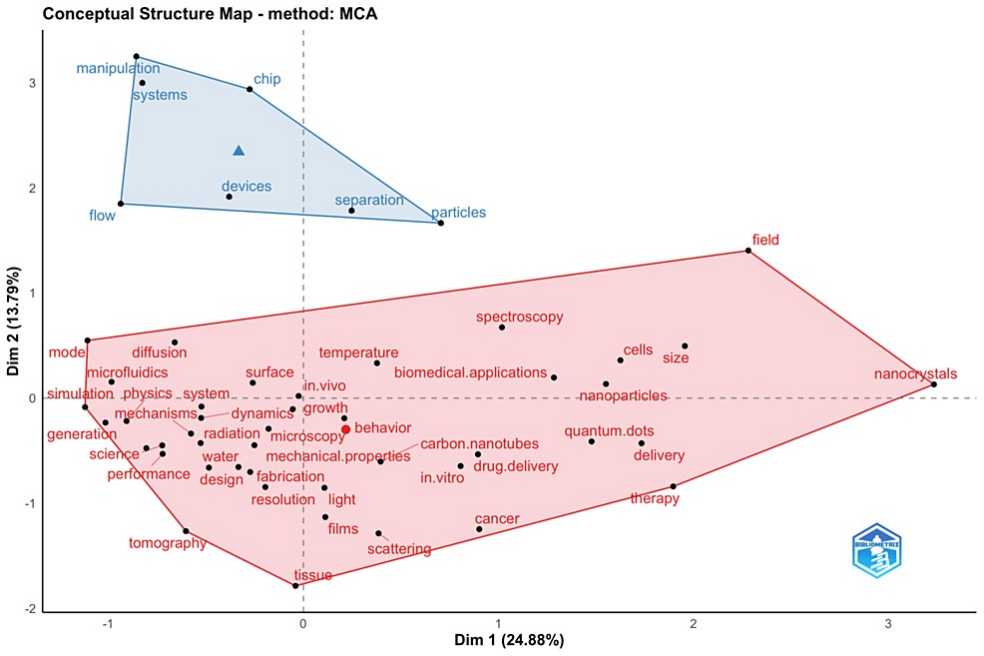
Factorial analysis of keywords

Figure 6 showcases a conceptual structure map using multiple correspondence analysis (MCA) to depict the relationship between various research themes [24]. The map is divided into quadrants along two dimensions that together account for a significant percentage of the inertia in the data, reflecting the major variations in the dataset. Dimension 1 (24.88%) could represent the degree of technological focus and application, stretching from basic physical principles like "diffusion" and "mechanics" on the left to advanced applications like "nanocrystals" and "therapy" on the right. Dimension 2 (13.79%) might illustrate the development stage, ranging from established methods such as "tomography" at the lower end to cutting-edge techniques like "manipulation" and "devices" at the upper end. The upper-right quadrant is characterized by contemporary and technologically advanced terms such as "nanoparticles," "quantum dots," and "drug delivery," which places a lot of the research effort in the context of the use of nanotechnology in therapy. This is in contrast to the lower left-hand quadrant where basic concepts like "tissue," "light," and "scattering" are placed, meaning that all those supposedly belong to the basic background of the discipline but might not represent the leading edge of current innovation. In the upper left-hand quadrant, which is high in the development degree but low in the relevance degree, one finds "flow" and "devices," probably referring to very developed but specific equipment and methodologies that are not central to the running mainstream. In general, the map offers a graphical representation of themes in research and their associated interrelations with saliency in a field. Furthermore, the strategic position in the map quadrants can provide guidance for researchers and policymakers in the identification of main knowledge areas, new and emerging technologies, and future research directions in biomedical physics.

Co-occurrence of Keywords Plus

**Figure 7:**
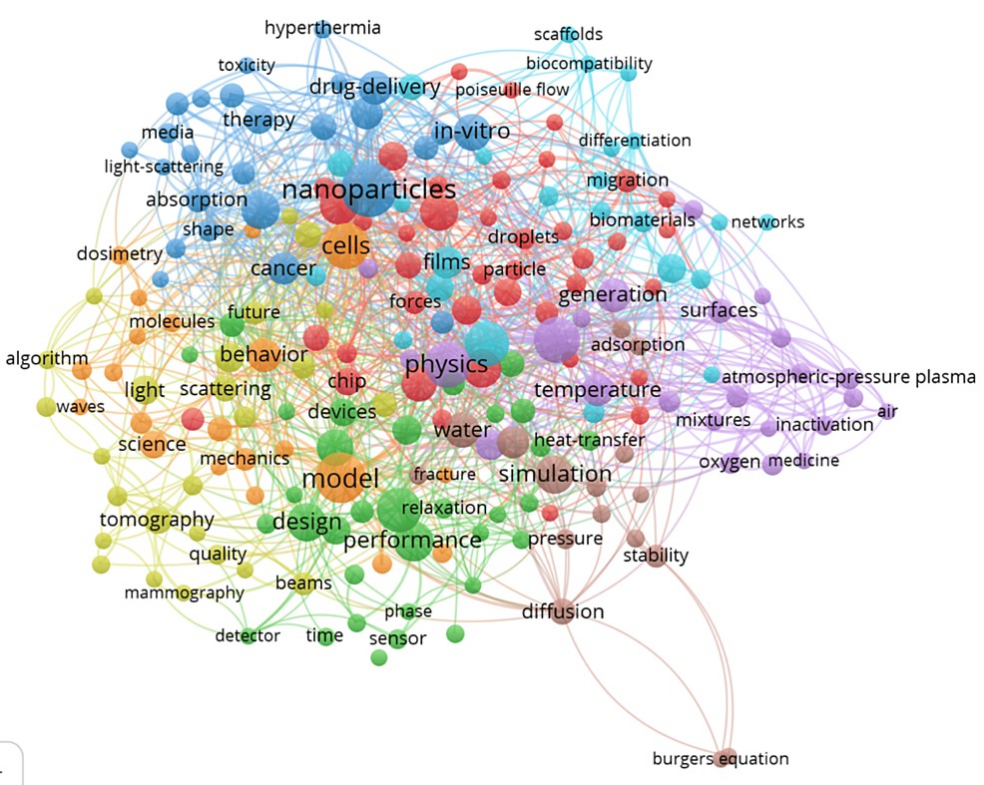
Co-occurrence of keyword plus

Figure 7 presents a multi-dimensional view of research themes with a co-occurrence map of keywords plus. The most prevalent keyword, "nanoparticles," is highlighted with 51 occurrences and a total link strength of 34, indicating its central role in the research network. It is likely part of Cluster 1, which is shown in red, denoting it as a primary area of focus within the field. This cluster possibly encompasses the development and application of nanoparticles in biomedical settings, given its strong connection with "biomedical applications," which has 34 occurrences and a total link strength of 15. Further scrutiny reveals "cells" with 34 occurrences and a link strength of 14, possibly situated in the green Cluster 2, symbolizing research into cellular interaction and therapies using nanotechnology. "Fabrication" (32 occurrences, 11 link strength) and "growth" (25 occurrences, 13 link strength) might be a part of the blue Cluster 3, suggesting a theme of synthesizing and growing biomedical materials. Keywords such as "model" (42 occurrences, 7 link strength) and "physics" (35 occurrences, 9 link strength) could belong to the yellow Cluster 4, indicating a concentration on theoretical and computational models that are integral to understanding the physical principles in biomedical applications. Finally, "particles" and "separation," both with 24 occurrences and link strengths of 14 and 9, respectively, may be a part of other clusters, reflecting the importance of particle dynamics and separation techniques in the study of biomedical physics.

Bibliographic Coupling of Countries

**Figure 8:**
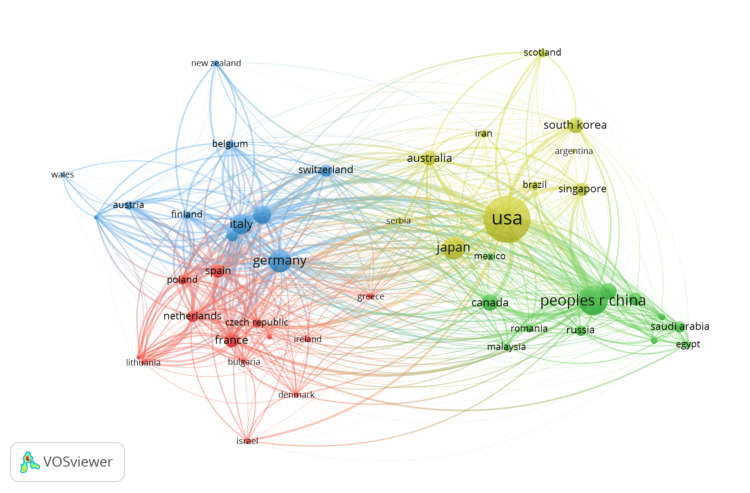
Bibliographic coupling of countries

Figure 8 presents the bibliographic coupling of countries and showcases the interconnected research output and collaborations among countries, using a VOSviewer network map to display the relationships based on shared citations. Four clusters, each distinguished by color, represent groupings of countries with similar research profiles or collaboration patterns in biomedical physics. The United States emerges as a central node within the red Cluster 1, with 428 documents and an extensive link strength of 8519, indicating its leading position in the research domain. Similarly, the People's Republic of China, prominent in the green Cluster 2, demonstrates considerable impact with 172 documents and a high citation count of 10,816. In the blue Cluster 3, Germany's significant activity is marked by 100 documents and a total link strength of 5,548, highlighting the country's strong research ties within the European region. Cluster 4, represented in yellow, includes countries like France and Italy, which contribute actively to the field, as evidenced by their substantial document counts and citations. This visualization, focusing on 46 influential countries out of a possible 76, illustrates the dense network of international collaborations and the flow of knowledge within biomedical physics, with the US and China at the forefront of research influence and Europe maintaining a solid presence through robust scholarly contributions and connections.

Co-citation of Cited Authors

**Figure 9:**
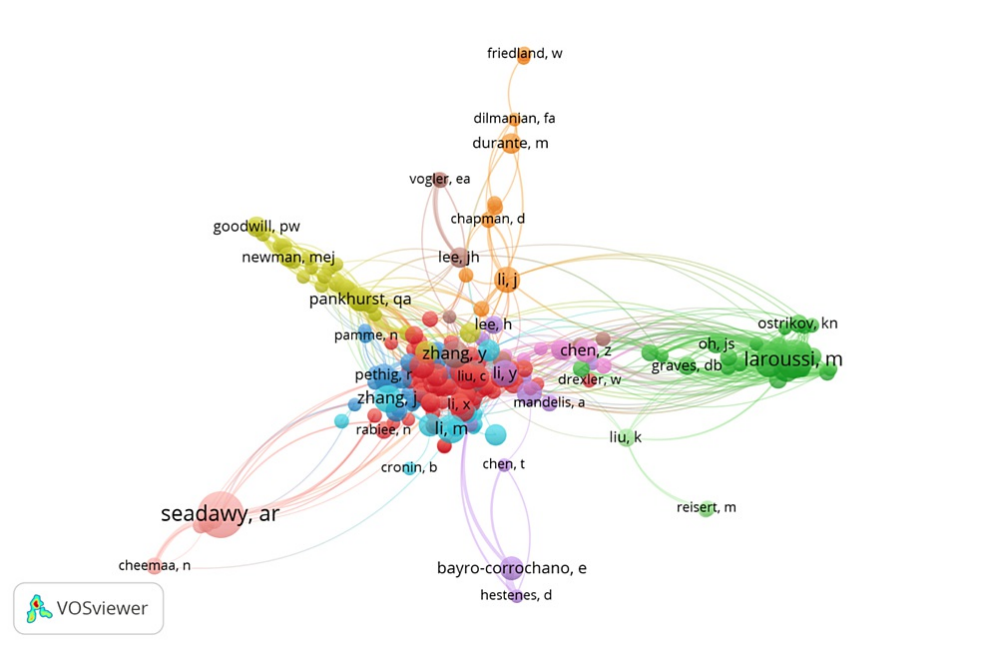
Co-citation of cited authors

The co-citation analysis of the cited authors within the study presented in Figure 9 demonstrates how frequently authors are cited together within a body of literature. The map consists of 11 clusters, each differentiated by color, signifying the interconnected scholarly networks and thematic concentrations tied to individual researchers' work. The map reflects the co-citation relationships among authors who meet a minimum citation threshold of 10, with 229 out of 35,733 authors fulfilling this criterion, focusing on the most impactful individuals in the field. Cluster 1, the largest with 74 authors and shown in red, may represent a central thematic area or a core group of highly connected and influential researchers. "Seadawy, AR" leads in this cluster with 109 citations but an atypical total link strength of 0, which could indicate a strong individual influence or a focal citation pattern that does not extensively overlap with others. In the green Cluster 2, comprising 28 authors, "Laroussi, M" is a notable contributor with 73 citations and a total link strength of 551, illustrating his central position within this sub-network of biomedical physics research. The other clusters, descending in size, indicate progressively more specialized or emerging areas of research, with Clusters 10 and 11 including authors who, despite fewer citations, are still significant within their niches. This co-citation network encapsulates the diverse and multifaceted nature of biomedical physics, highlighting the authors whose work forms the backbone of the field's scholarly dialogue. It serves as a testament to the collaborative spirit of scientific inquiry, where the impact and relevance of research are not just in the findings but also in the ongoing conversations and connections they foster within the academic community.

Research gaps and practical implications

Trend topics and thematic maps in this analysis reveal evolving research focus areas and uncover potential research gaps and implications. Initially, the field concentrated on foundational topics such as "microfluidics," "mechanics," and "image reconstruction," which are essential for developing methods to manipulate and visualize biological processes. This phase highlights an early research gap in the integration of these techniques with clinical applications, where further work could optimize these foundational technologies for direct patient care and diagnostics.

As the timeline progresses, there's a marked shift toward materials science and nanotechnology, with a significant uptick in research on "nanoparticles," "carbon nanotubes," and "magnetic nanoparticles." This transition underscores a pivotal research gap in understanding the long-term biocompatibility and safety of nanomaterials in biomedical applications, an area ripe for extensive study. Moreover, the recent emphasis on "biomedical applications" signals a maturation of the field but also points to a gap in translating laboratory-scale innovations to scalable clinical solutions. The practical implications of this phase involve enhancing the translational research infrastructure to bridge the gap between nanotechnology innovations and their practical, regulatory-approved medical applications.

In the last few years, technological developments in the surgical field have been rapid and are continuously evolving. One of the most revolutionary breakthroughs was the introduction of the Internet of Things (IoT) concept within medical practice [25]. This integration has significantly enhanced the capability of medical devices to communicate and collaborate, leading to improved operational efficiency and patient outcomes. The relationship between IoT and biomedical physics is particularly significant, as it bridges the gap between physical principles and their application in real-time medical scenarios. Biomedical physics, which focuses on applying the concepts of physics to medicine, finds a complementary technological framework in IoT. This integration allows for real-time monitoring and analysis of physiological data, which is crucial in surgical settings and therapeutic interventions. IoT-enabled devices can collect and transmit data continuously, allowing biomedical physicists to apply their understanding of physical processes in diagnosing, treating, and monitoring patients more effectively. For instance, in radiation therapy, IoT can facilitate the precise delivery of treatment by synchronizing equipment and patient data, thus enhancing the application of medical physics principles in oncology. Moreover, IoT contributes to the development of "smart" healthcare environments where biomedical physics principles can be applied more efficiently to achieve personalized medicine. Through the continuous feedback provided by IoT systems, medical physicists can optimize clinical protocols and procedures in real time, tailoring them to individual patient needs based on accurate physical data analysis.

In the latest years, the focus on refining "ultrasound," "contrast agents," and "radiography" indicates ongoing advancements in imaging technologies. This suggests a continuous need for research that enhances the resolution, specificity, and safety of imaging techniques, especially in non-invasive diagnostics. Future research could explore the integration of artificial intelligence to improve the accuracy and predictive power of imaging systems, addressing a critical research gap in real-time, personalized diagnostic processes. Overall, the bibliometric analysis not only maps the historical shifts in biomedical physics but also directs future research efforts toward closing critical gaps and harnessing emerging technologies to meet evolving healthcare demands.

One significant limitation of this study is the restricted use of the keyword "biomedical physics," which may have narrowed the scope of our review. The field encompasses a wide range of sub-disciplines, including medical physics, biophysics, bioengineering, and many others. By focusing narrowly, we may have overlooked substantial contributions from these related areas, potentially missing key authors and studies with significant relevance to our topic. Future research could benefit from a broader keyword selection to capture the full diversity of the field and ensure a more comprehensive analysis of the literature.

## Conclusions

The bibliometric analysis of biomedical physics has provided valuable insights into the developmental trajectory and current landscape of this vital interdisciplinary field. Our study has revealed consistent growth in scientific output, reflecting the ongoing relevance and expansion of biomedical physics in addressing complex healthcare challenges. The identification of key authors and influential journals has highlighted central nodes of knowledge and dissemination within the community. Through thematic and factorial analyses, we have pinpointed emerging trends and the integration of new technologies, such as nanotechnology and advanced imaging techniques, which are setting the direction for future research. Based on our findings, we recommend increased funding and support for research in emerging areas like nano-scale drug delivery systems and non-invasive imaging technologies, which hold significant promise for transforming patient care. Furthermore, fostering collaborative networks that bridge gaps between physicists, biologists, and medical professionals can enhance innovation and application of research findings. Lastly, there is a clear need for developing educational programs that focus on the interdisciplinary skills required in biomedical physics to prepare the next generation of researchers to continue advancing this crucial field.
